# The proportion of the population of England that self-identifies as lesbian, gay or bisexual: producing modelled estimates based on national social surveys

**DOI:** 10.1186/s13104-017-2921-1

**Published:** 2017-11-13

**Authors:** Sanne Christine van Kampen, William Lee, Mauro Fornasiero, Kerryn Husk

**Affiliations:** Clinical Trial and Population Studies Department, Plymouth University Peninsula Schools of Medicine and Dentistry, 1 Davy Road, Plymouth, PL6 8BX UK

**Keywords:** Demography, Geography, Health inequality, Modelling, Systematic review

## Abstract

**Objectives:**

There is currently no widely accepted estimate of the proportion of people in England that self-identifies as lesbian, gay or bisexual (LGB), which is needed if we are to compare health inequality between different population groups. Using systematic review methods, this study identified all national social surveys with a question on sexual orientation and pooled those which represented the overall population of England. LGB proportions were synthesized into an aggregated mean estimate using weights based on sample size, response rate and missing data. The modelled estimate was stratified by socio-demographic and geographical variables.

**Results:**

Twenty-two national surveys were identified of which 15 were suitable for pooling. Synthesis resulted in a weighted mean estimate of 2.50% of the adult population of England identifying as LGB or ‘other’. The proportion was highest in men, people below 45 years of age and the London region. The (theoretical) upper limit was 5.89% if all non-responders were assumed to identify as LGB. The reported 2.50% presents a minimum and may be influenced by respondents’ perceptions of confidentiality and social acceptance. It is however the most robust estimate currently available and can be used as baseline to understand health and wellbeing needs of different groups.

**Electronic supplementary material:**

The online version of this article (10.1186/s13104-017-2921-1) contains supplementary material, which is available to authorized users.

## Introduction

Sexual orientation is a protected characteristic under the UK Equality Duty of the Equality Act 2010 [1], so public bodies must have due regard for the need to reduce discrimination and advance equal opportunities among those who share such protected characteristics and those who do not. Furthermore, it is important for public health bodies to understand the health and wellbeing needs of minority sexual orientation groups, as they are known to be at increased risk of poor physical and mental health behaviours and outcomes [2–4]. An important first step in discharging this responsibility is to know the proportion of people who self-identify as lesbian, gay or bisexual (LGB) in England. Unfortunately there is currently no agreed and supported estimate available.

The authors were commissioned by Public Health England to devise a process to use all available data to derive the best possible estimate of the proportion of people in England who self-identify as LGB. In 2009, the Office of National Statistics (ONS) developed a standard question to ask sexual identity on social surveys, which was subsequently introduced in a number of national questionnaires [5–7]. So far, no study has used a systematic approach to identify all surveys that measure sexual orientation and synthesise them taking methodological limitations into account. This study aimed to produce a robust estimate of the proportion of people who self-identify as LGB, which could be used as a baseline figure for researchers and policy makers to compare health inequality and inequity between different population groups.

## Main text

### Methods

First, nine relevant databases (EMBASE, HSCIC, MEDLINE, SAGE, Social Care Online, Social Science Research Network, SocINDEX, UK Data Archive, Web of Science) were searched using a combination of search terms, e.g. in Pubmed/MEDLINE: (“sexual orientation” OR “sexual identity” OR “same-sex relationships” or “lesbian gay bisexual”) AND (“United Kingdom” OR England OR Britain) AND (survey OR questionnaire OR proportion OR prevalence OR size OR percentage OR measure OR estimate) (see Additional file 1). In addition, the grey literature was searched by exploring websites from key organizations (National Health Services, ONS, Stonewall, LGBT Foundation), hand-searching publications and exploiting author-contacts. The survey inclusion criteria were: (1) geographical coverage of at least the whole of England or sub-geographies that together form a representative sample of the whole of England; (2) targeting the general population or a sub-set of the general population unlikely to affect sexual identity proportions; and (3) including a direct question on a person’s sexual identity. There was no time restriction, but only the most recent version of a survey series or longitudinal cohort was included.

Second, from each identified survey, methodological data were extracted including; geographical coverage, data collection period, survey population, survey design, sampling method, sample size and response rate. With regard to the sexual orientation question, data were extracted on; question format, mode of administration, response categories and proportions. Response categories included both substantive answers (heterosexual/straight; lesbian/gay; bisexual; other) and non-substantive answers (don’t know, prefer not to say, refused, no answer). Individual survey proportions of LGB people were calculated as the sum of the proportions of ‘gay/lesbian’, ‘bisexual’ and ‘other’ among all those who were asked the question on sexual orientation. The responses were limited to the population of England.

Third, a quality assessment was done to determine which surveys to pool into a synthesized estimate and what weights to assign to each survey in a synthesis. Surveys with study populations that did not represent the general population of England in terms of age, gender or other characteristics may introduce bias in LGB proportions and were therefore not pooled but reported separately [8, 9]. For the synthesis we used an adaptation of a previously developed method to enumerate minority ethnic groups from surveys [10, 11]. Weights were assigned to methodological characteristics that differed between surveys, were conceptually linked to sexual orientation response quality and were quantifiable, which were: survey sample size; overall survey response rate; and proportion of missing data. To avoid overweighting we used a logarithmic transformation of sample size. We used an inverse proportion of all non-substantive answers (don’t know, prefer not to say, refused, no answer) as a weight for missing data. Five different combinations of weights were used to explore their relative effects (see Additional file 2). The fifth method incorporated all three weights and was considered to be the most robust method. A range was constructed around the aggregated mean estimate by performing a sensitivity analysis around missing data, calculating the most extreme scenarios where people with non-substantive answers would have been either all heterosexual or all lesbian/gay/bisexual.

Finally, we stratified the mean estimate by age, gender and region. Since LGB proportions could not be stratified for all original surveys, we selected a baseline survey to provide a standard distribution of LGB. Ideally, the Census of England and Wales 2011 would have been used for this purpose, but this survey did not include a question on sexual orientation [12]. We found that the GP (general practitioner) Patient Survey 2015 best resembled the population of England in terms of age, gender and region. Using the distribution of LGB across strata from the GP Patient Survey and our synthesized mean LGB estimate, we calculated the estimated number of adult LGB people in England in 2015 and divided this by the total population numbers based on mid-2015 ONS’ estimates [13].

### Results

We identified a total of 664 records; 617 from published data sources and 47 from grey sources. Of these, 636 were excluded because they did not meet the inclusion criteria. After full-text screening of the remaining 28 surveys, six more were excluded: four were previous versions of more recent surveys already included and two surveys formed part of an umbrella survey (Integrated Household Survey 2014) which was already included. The remaining 22 surveys were similar in terms of study design, question format and substantive response categories, while differences were found in terms of study populations, sampling methods, sample sizes, survey response rates, modes of question administration and non-substantive response categories (Additional file 3).

**Fig. 1:**
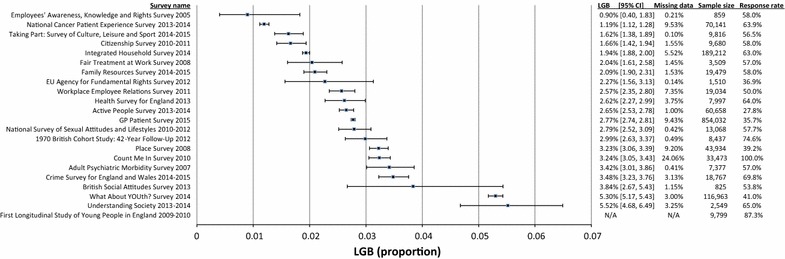
Proportion of survey participants who self-reported as lesbian, gay, bisexual or ‘other’. This figure shows the results of 22 National Social Surveys that included a question on sexual identity. For each survey, it provides the name, data collection period, proportion (filled square) and 95% confidence interval (horizontal brackets) of people who self-identified as lesbian, gay, bisexual or other. In addition, proportion of missing data as well as the overall survey sample size and response rate are shown. Missing data represent all people who responded ‘don’t know’, ‘prefer not to say’, refused or gave no answer to the sexual orientation question. For the Family Resources Survey, the LGB estimate is unweighted, because the weighted proportions were less precise than the unweighted proportions. For the Count Me In Survey; National Cancer Patient Experience Survey; and 1970 British Cohort Study, the LGB estimates are unweighted, because the surveys sampled the entire target population. For the EU Agency for Fundamental Rights—Violence Against Women Survey, the LGB estimate is for the response category of ‘non-heterosexual’. This survey made no differentiation between lesbian/gay, bisexual and other. The data is for the UK and could not be specified for England. For the British Social Attitudes Survey, the estimate is for the response categories ‘gay’, ‘bisexual’ and ‘can’t choose’. This survey had no category for ‘other’. For the Count Me In Survey, the proportion of ‘no answers’ was very high, while the survey response rate could not be retrieved. It is therefore likely that at least a proportion of people with ‘no answer’ were in fact not eligible to respond or never asked the question. For the First Longitudinal Study of Young People in England survey, we were not able to obtain original data

The proportions of LGB and ‘others’ among people who were asked the question on sexual orientation in the 22 surveys are shown in Fig. 1. Percentages ranged from 0.90% (95% CI 0.40, 1.83) to 5.52% (95% CI 4.63, 6.56). The proportion of missing data ranged from 0.10 to 24.06%. Sample sizes ranged from 825 to 854,032 and response rates from 28 to 100%. The results of one survey, the First Longitudinal Study of Young People in England: Waves 1–7 2009–2010, could not be obtained.

The following seven surveys were excluded from pooling because their study population was limited in terms of age, gender or health conditions:

- 1970 British Cohort Study: 42-year follow-up 2012.
- Health and Wellbeing of 15 year olds in England—What About YOUth? Survey 2014.
- First Longitudinal Study of Young People in England: Waves 1–7 2009–2010.
- Understanding Society: Waves 1–5 (‘UK Household Longitudinal Study’) 2013–2014.
- EU Agency for Fundamental Rights: Violence Against Women Survey 2012.
- National Cancer Experience Survey 2013–2014.
- Count Me In Survey 2010 (patients of mental health services).

Thus, 15 of the 22 surveys were suitable for pooling and their measures of sexual orientation were synthesized using the five different weighting methods described above (Table 1).

**Table 1:** Estimates of the size of the lesbian, gay and bisexual (LGB) population of England

Method	Weighting approach	LGB (%)	Lesbian and gay (%)	Bisexual (%)	‘Other’ (%)
1	Unweighted	2.51	1.25	0.60	0.65
2	Weighted by log sample size	2.52	1.27	0.63	0.62
3	Weighted by log sample size and response rate	2.51	1.26	0.63	0.61
4	Weighted by inverse proportion of missing data^a^	2.50	1.25	0.60	0.66
5	Weighted by log sample size, response rate and inverse proportion of missing data^a^	2.50	1.25	0.63	0.61

^a^Missing data: people who responded ‘don’t know’, ‘prefer not to say’, refused and gave no answer

Ranges around the mean LGB estimate of 2.50% (Method 5) resulted in a minimum of 2.50% and maximum of 5.89%, when people who responded ‘prefer not to say’, ‘refused’, ‘don’t know’ or ‘no answer’ were assumed to be either all heterosexual (lower bound) or all LGB (upper bound). The aggregated mean estimate was subsequently stratified by age, gender and government region based on the distribution of LGB of the broadest survey (the GP Patient Survey 2015) (Table 2). Applied to the adult population of England in mid-2015, the proportion of LGB and ‘other’ was highest among young adults from 18 to 34 (3.74%) and decreased with each older age group. The proportion was higher in men (3.13%) than women (1.90%), which is consistent with findings from other surveys, including the Integrated Household Survey 2014, Health Survey for England 2013, British Social Attitudes Survey 2013, Taking Part Survey 2014–2015 and the Active People Survey 2013–2014. The proportion of LGB was highest in the London region (4.29%), while it was around 2.0–2.5% in the other regions of England.

**Table 2:** Stratified estimates of the lesbian, gay and bisexual (LGB) adult population of England

	GP Patient Survey 2015		Estimated population of England mid-2015	Projections to population of England mid-2015	
	Number of LGB	Distribution of LGB (%)		Number of LGB	Distribution of LGB (%)
Age (years)					
0–17^a^	–	–	11,677,856	–	–
18–24	4030	17.1	4,920,128	184,027	3.74
25–34	6122	25.9	7,485,996	279,556	3.73
35–44	5161	21.9	7,107,372	235,673	3.32
45–54	4266	18.1	7,700,360	194,804	2.53
55–64	2099	8.9	6,183,043	95,849	1.55
65–74	1075	4.6	5,285,755	49,089	0.93
75–84	581	2.5	3,130,528	26,531	0.85
85 or over	283	1.2	1,295,289	12,923	1.00
Total	23,617	100.0	43,108,471	1,078,452	2.50
Gender					
Male	14,434	61.1	21,047,349	659,173	3.13
Female	9181	38.9	22,061,122	419,279	1.90
Total	23,615	100.0	43,108,471	1,078,452	2.50
Region					
London	6482	26.8	6,720,843	288,531	4.29
North West	3197	13.2	5,652,470	142,307	2.52
North East	1060	4.4	2,100,204	47,183	2.25
South East	3524	14.5	7,029,838	156,863	2.23
West Midlands	2187	9.0	4,489,117	97,349	2.17
Yorkshire and the Humber	1981	8.2	4,244,933	88,180	2.08
South West	2012	8.3	4,389,099	89,559	2.04
East of England	2148	8.9	4,776,467	95,613	2.00
East Midlands	1637	6.8	3,705,500	72,867	1.97
Total	24,228	100.0	43,108,471	1,078,452	2.50

^a^The proportion of LGB among people below 18 could not be estimated as the GP Patient Survey only asked about sexual orientation among adults (18+)

### Discussion

This study provides an aggregated weighted estimate of the size of the LGB (and ‘other’) population of England of 2.50% with a range of 2.50–5.89% based on a sensitivity analysis of missing data. This would project to an estimated 1.08 million adults self-identifying as belonging to a sexual minority among a total of 43.1 million people in 2015 (ONS mid-2015 estimates). The upper bound of 5.89% should be treated with caution as it represents the theoretical maximum if *all people who did not respond informatively to a question on sexual orientation would report as LGB*. The aggregated mean of 2.50% provides the lowest possible estimate of LGB in the given sources, which is slightly lower than that of national household surveys of the United States, Canada and Australia, where figures have been reported between 2.4–3.5, 3.0 and 3–4%, respectively [14–18]. This is the first study to adopt a systematic and weighting approach to identify and combine the results of existing surveys into an estimate of the LGB population of England. Both the search strategy and synthesis methodology were discussed with a group of experts in the field of sexual orientation surveys in England. As a result, we are confident that all relevant surveys are included and that the methodology is robust. However, our LGB estimates are clearly sensitive to problems in the original surveys and societal factors relating to reporting of sexual orientation.

## Limitations

Our study had several limitations:

- The synthesis included weights based on survey sample size, response rate and proportion of missing data. Yet other factors may also have influenced non-response and misreporting of sexual orientation, including mode of question administration and survey context. However, given the lack of knowledge about the direction and magnitude of these effects, the current methodology could not include quantitative weights for them. We also did not include a weight for variance as is usual in meta-analysis, as we felt it was more important to use weights conceptually linked to problems of reporting sexual orientation rather than weights reflecting precision.
- The stratified aggregated LGB estimates were valid only to the extent to which the population distributions of the baseline survey (the GP Patient Survey 2015) were representative of the national population of England. While the distributions of age, gender and region were very similar between the two, there was variation in ethnicity which meant that we could not confidently report estimates stratified by ethnicity. Using the GP Patient Survey as our baseline survey also meant that we were not able to stratify by local authority level or disability, because the survey simply did not provide this type of information. These issues could be resolved if original surveys would stratify their results by these factors or if the Census would include a question on sexual orientation [19]. It would also be useful to have more surveys conducted at local level to get a better understanding of geographical differences.
- Using general population surveys to quantify the proportion of people who self-identify as LGB may have underestimated this group, because some people may inaccurately report their sexual identity in survey settings influenced by perceptions of confidentiality and social acceptance [20–22]. While these issues may change slowly over time, future surveys may be able to produce more reliable and realistic estimates if the context and mode of administration of sexual identify questions is optimized. Also, social acceptance may increase if the question is adopted in the national Census. Finally, it is important to acknowledge that sexual identity as used in national surveys is not coterminous with sexual orientation, which is the term used in the Equality Act to legally protect LGB people from discrimination, and that any of these estimates therefore are likely to underestimate the actual size of this population.

## Additional files

**Additional file 1.** Search strategies run between 25 February and 9 March 2016. List of nine databases that were searched with search terms and number of articles retrieved.

**Additional file 2.** Formulas to synthesize survey proportions into weighted aggregated estimates. Five different methods and formulas used to calculated weighted averages of survey proportions of people who self-identified as lesbian, gay or bisexual.

**Additional file 3.** Methodological characteristics of 22 surveys that included a question on sexual orientation. List of 22 included surveys with their name, data collection period, geographical coverage, study population, sampling method, sample size, response rate and mode of administration.

## Abbreviations

GP: general practitioner
LGB: lesbian, gay and bisexual
ONS: Office for National Statistics

## References

[1] Equality Act. Stat. Chapter 15. London: UK Government; 2010. http://www.legislation.gov.uk/ukpga/2010/15/pdfs/ukpga_20100015_en.pdf. Accessed June 2016.

[2] King M, Semlyen J, Tai SS, Killaspy H, Osborn D, Popelyuk D (2008). A systematic review of mental disorder, suicide, and deliberate self harm in lesbian, gay and bisexual people. BMC Psychiatry.

[3] Semlyen J, King M, Varney J, Hagger-Johnson G (2016). Sexual orientation and symptoms of common mental disorder or low wellbeing: combined meta-analysis of 12 UK population health surveys. BMC Psychiatry.

[4] Meads C, Carmona C, Kelly MP (2012). Lesbian, gay and bisexual people’s health in the UK: a theoretical critique and systematic review. Divers Equal Health Care.

[5] Purdam K, Wilson AR, Afkhami R, Olsen W (2008). Surveying sexual orientation: asking difficult questions and providing useful answers. Cult Health Sex.

[6] Haseldon L, Joloza T (2009). Measuring sexual identity: a guide for researchers.

[7] Office for National Statistics (2008). Developing survey questions on sexual identity: rationale and design of sexual identity questioning on the Integrated Household Survey (IHS).

[8] Joloza T, Evans J, O’Brien R, Potter-Collins A (2010). Measuring sexual identity: an evaluation report.

[9] Statistical bulletin: Integrated Household Survey (Experimental statistics): January to December 2014. Newport: Office for National Statistics; 2015. http://www.ons.gov.uk/peoplepopulationandcommunity/culturalidentity/sexuality/bulletins/integratedhouseholdsurvey/2015-10-01. Accessed June 2016.

[10] Husk K (2012). Cornish ethnicity and undercounting: utilising the 2001 England and Wales Census to develop an accurate measurement methodology. Methodol Innov Online.

[11] Husk K (2012). The legitimation of ethnicity: the case of the Cornish. Stud Ethn Nationalism.

[12] The 2011 census: assessment of initial user requirements on content for England and Wales—sexual orientation. Newport: Office for National Statistics; 2006. https://www.ons.gov.uk/census/2011census/consultationsusersandlocalpartners/2011censusclosedconsultations/consultationon2011censusresponses. Accessed June 2016.

[13] Office for National Statistics. Population estimates analysis tool. Mid-2015. Newport: Office for National Statistics; 2016. https://www.ons.gov.uk/peoplepopulationandcommunity/populationandmigration/populationestimates/datasets/populationestimatesanalysistool. Accessed April 2017.

[14] US Department of Health & Human Services. National Health Interview Survey. Sexual Orientation Information. Atlanta. 2015. https://www.cdc.gov/nchs/nhis/sexual_orientation/index.htm. Accessed June 2016.

[15] Gates G, Newport F. LGBT percentage highest in D.C., lowest in North Dakota: Gallup; 2013. http://www.gallup.com/poll/160517/lgbt-percentage-highest-lowest-north-dakota.aspx. Accessed June 2016.

[16] Gates GJ. How many people are lesbian, gay, bisexual and transgender? Los Angeles: Williams Institute: UCLA; 2011. http://williamsinstitute.law.ucla.edu/wp-content/uploads/Gates-How-Many-People-LGBT-Apr-2011.pdf. Accessed June 2016.

[17] Canadian Community Health Survey. Same-sex couples and sexual orientation by the numbers. Ottawa: Statistics Canada; 2014. http://www.statcan.gc.ca/eng/dai/smr08/2015/smr08_203_2015#a3. Accessed Jan 2017.

[18] Richters J, Altman D, Badcock PB, Smith AMA, DeVisser RO, Grulich AE (2014). Sexual identity, sexual attraction and sexual experience: the Second Australian Study of Health and Relationships. Sex Health.

[19] Botcherby S, Creegan C (2009). Moving forward: putting sexual orientation in the public domain.

[20] Ellison G, Gunstone B. Sexual orientation explored: a study of identity, attraction, behaviours and attitudes in 2009. In: YouGov, editor. Manchester: Equality and Human Rights Commission; 2009. ISBN 978 1 84206 224 1.

[21] Berg N, Lien D (2006). Same-sex sexual behaviour: US frequency estimates from survey data with simultaneous misreporting and non-response. Appl Econ.

[22] Copas AJ, Wellings K, Erens B, Mercer CH, McManus S, Fenton KA (2002). The accuracy of reported sensitive sexual behaviour in Britain: exploring the extent of change 1990–2000. Sex Transm Infect.

