# Supplementary material for: The proportion of the population of England that self-identifies as lesbian, gay or bisexual: producing modelled estimates based on national social surveys
Source: BMC Res Notes. 2017 Nov 13;10:594. doi: 10.1186/s13104-017-2921-1 (PMC5683336; doi:10.1186/s13104-017-2921-1)
Supplement: Supplementary file 1 — Additional file 1. Search strategies run between 25 February and 9 March 2016. List of nine databases that were searched with search terms and number of articles retrieved. [file 13104_2017_2921_MOESM1_ESM.docx]

**Additional file 1**

Search strategies run between 25 February and 9 March 2016

| **Database** | **Search terms** | **Total articles retrieved** |
| --- | --- | --- |
| EMBASE | ("sexual orientation” OR “sexual identity” OR “same-sex relationships” or “lesbian gay bisexual”) AND (UK OR England) AND (survey OR questionnaire OR proportion OR prevalence OR size OR percentage) | 91 |
| HSCIC | (‘sexual orientation survey’) OR (‘sexual identity survey’) | 68 |
| Pubmed/ MEDLINE | ("sexual orientation” OR “sexual identity” OR “same-sex relationships” or “lesbian gay bisexual”) AND (“United Kingdom” OR England OR Britain) AND (survey OR questionnaire OR proportion OR prevalence OR size OR percentage OR measure OR estimate) | 106 |
| SAGE | "sexual orientation” AND England AND survey | 41 |
| Social Care Online | "sexual orientation” AND survey AND “United Kingdom” | 5 |
| Social Science Research Network | "sexual orientation” AND survey | 38 |
| SocINDEX | ("sexual orientation” OR “sexual identity” OR “same-sex relationships” or “lesbian gay bisexual”) AND (“United Kingdom” OR England OR Britain) AND (survey OR questionnaire OR proportion OR prevalence OR size OR percentage OR measure OR estimate) | 69 |
| UK Data Archive ^1^ | ("sexual orientation" OR "sexual identity" OR "same-sex relationships" OR "lesbian gay bisexual") AND (England OR "United Kingdom" OR "Great Britain" OR "England and Wales") | 89 |
| Web of Science | ("sexual orientation” OR “sexual identity” OR “same-sex relationships” or “lesbian gay bisexual”) AND (“United Kingdom” OR England OR Britain) AND (survey OR questionnaire OR proportion OR prevalence OR size OR percentage) | 97 |

^1^ The UK Data Archive was searched using both ‘Discovery’ and ‘Variable and Question Bank’.
