# Supplementary material for: The proportion of the population of England that self-identifies as lesbian, gay or bisexual: producing modelled estimates based on national social surveys
Source: BMC Res Notes. 2017 Nov 13;10:594. doi: 10.1186/s13104-017-2921-1 (PMC5683336; doi:10.1186/s13104-017-2921-1)
Supplement: Supplementary file 2 — Additional file 2. Formulas to synthesize survey proportions into weighted aggregated estimates. Five different methods and formulas used to calculated weighted averages of survey proportions of people who self-identified as lesbian, gay or bisexual. [file 13104_2017_2921_MOESM2_ESM.docx]

**Additional file 2**

Formulas to synthesize survey proportions into weighted aggregated estimates:

*Method 1.* Simple aggregated mean (no weighting):

$\frac{\left( \text{p}\text{1+}\text{p}\text{2+…+}\text{pk} \right)}{\text{k}}$

*Method 2.* Aggregated mean weighted by log sample size:

$\frac{\left( p1\times s1 \right)+\left( p2\times s2 \right)+\ldots+\left( pk\times sk \right)}{s1+s2+\ldots+sk}$

*Method 3.* Aggregated mean weighted by log sample size and survey response rate: $\frac{\left( p1\times s1\times r1 \right)+\left( p2\times s2\times r2 \right)+\ldots+\left( pk\times sk\times rk \right)}{\left( s1\times r1 \right)+\left( s2\times r2 \right)+\ldots+\left( sk\times rk \right)}$

*Method 4.* Aggregated mean weighted by inverse proportion of missing data: $\frac{\left( p1\times w1 \right)+\left( p2\times w2 \right)+\ldots+\left( pk\times wk \right)}{w1+w2+\ldots+wk}$

*Method 5.* Aggregated mean weighted by log sample size, response rate and missing data: $\frac{\begin{aligned} \left( p1\times s1\times r1\times w1 \right)+\left( p2\times s2\times r2\times w2 \right)+\ldots\\ +\left( pk\times sk\times rk\times wk \right) \end{aligned}}{\left( s1\times r1\times w1 \right)+\left( s2\times r2\times w2 \right)+\ldots+\left( sk\times rk\times wk \right)}$

Legend. p=proportion LGB and ‘other’; k=number of surveys; s=logarithmic transformation of sample size; r=response rate (proportion); and w=weight for missing data= 1- (proportion don’t know + prefer not to say + refused + no answer)
