# Supplementary material for: The proportion of the population of England that self-identifies as lesbian, gay or bisexual: producing modelled estimates based on national social surveys
Source: BMC Res Notes. 2017 Nov 13;10:594. doi: 10.1186/s13104-017-2921-1 (PMC5683336; doi:10.1186/s13104-017-2921-1)
Supplement: Supplementary file 3 — Additional file 3. Methodological characteristics of 22 surveys that included a question on sexual orientation. List of 22 included surveys with their name, data collection period, geographical coverage, study population, sampling method, sample size, response rate and mode of administration. [file 13104_2017_2921_MOESM3_ESM.docx]

Additional file 3

**Methodological characteristics of 22 surveys that included a question on sexual orientation**

| **Survey Name** | **Collec-tion period** | **Geogra-phy ^a^** | **Study population** | | **Sampling method** | | | |  | |  | |  |
| --- | --- | --- | --- | --- | --- | --- | --- | --- | --- | --- | --- | --- | --- |
|  |  |  | **Age** | **Group** | **Sam-pling frame ^b^** | **Stratification level** | **Sample size** | **Sample of England** | | **Res-ponse rate** | | **Mode of admin ^c^** | |
| Integrated Household Survey | 2014 | UK | 16+ | private households & student halls | small user PAF | simple | 258,661 | 189,212 | | 63.0% | | SHOWTEL | |
| National Survey of Sexual Attitudes and Lifestyles | 2010 2011 2012 | GB | 16-74 | private households | small user PAF | complex | 15,162 | 13,068 | | 57.7% | | SHOW | |
| Health Survey for England | 2013 | ENG | 16+ | private households | small user PAF | complex | 7,997 | 7,997 | | 64.0% | | PAP | |
| Crime Survey for England and Wales | 2014 2015 | ENG, WAL | 16-59 | private households | small user PAF | complex | 20,908 | 18,767 | | 69.8% | | CASI | |
| Adult Psychiatric Morbidity Survey | 2007 | ENG | 16+ | private households | small user PAF | complex | 7,377 | 7,377 | | 57.0% | | CASI | |
| Family Resources Survey | 2014 2015 | UK | 16+ | private households | small user PAF | complex | 27,120 | 19,479 | | 58.0% | | SHOW | |
| Place Survey | 2008 | ENG | 18+ | private households | small user PAF | complex | 43,934 | 43,934 | | 39.2% | | PAP | |
| Citizenship Survey | 2010 2011 | ENG, WAL | 16+ | private households | small user PAF | complex | 16,966 | 9,680 | | 58.0% | | SHOW | |
| British Social Attitudes Survey | 2013 | GB | 18+ | private households | small user PAF | complex | 966 | 825 | | 53.8% | | PAP | |
| Taking Part: the National Survey of Culture, Leisure and Sport | 2014 2015 | ENG | 16+ | private households | small user PAF | complex | 9,816 | 9,816 | | 56.5% | | SHOW | |
| Active People Survey | 2013 2014 | ENG | 16+ | private households with landline | Random Digit Dialling | simple | 60,658 | 60,658 | | 27.8% | | TEL | |
| GP Patient Survey | 2015 | ENG | 18+ | registered with general practitioner | HSCIC patient registration records | less complex | 854,032 | 854,032 | | 35.7% | | PAP WEB TEL | |
| Fair Treatment at Work Survey | 2008 | GB | 16+ | in employment | small user PAF | complex | 4,010 | 3,509 | | 57.0% | | CASI | |
| Workplace Employee Relations Survey | 2011 | GB | all | in employment | Inter Departmental Business Register | less complex | 21,981 | 19,034 | | 50.0% | | PAP WEB | |
| Employees' Awareness, Knowledge and Exercise of Employment Rights Survey | 2005 | GB | 16-64 (M) 16-59 (F) | in employment | small user PAF | complex | 1,038 | 859 | | 58.0% | | SHOW | |
| National Cancer Patient Experience Survey | 2013; 2014 | ENG | 16+ | patients treated for cancer | none | not applicable | 70,141 | 70,141 | | 63.9% | | PAP | |
| Count Me In | 2010 | ENG, WAL | all | patients mental health services | none | not applicable | 36,091 | 33,473 | | - | | - | |
| First Longitudinal Study of Young People in England: Waves 1-7 | 2009; 2010 | ENG | 18-19 | in school | Pupil Level Annual Schools Census | less complex | 9,799 | 9,799 | | 87.3% | | CASI TEL WEB | |
| Understanding Society: Waves 1-5 ('UK Household Longitudinal Study') | 2013; 2014 | UK | 16-21 | private households | small user PAF | complex | 3,354 | 2,549 | | 65.0% | | CASI TEL WEB | |
| Health and Wellbeing of 15 year olds in England – What About YOUth? Survey | 2014 | ENG | 15 | in school | National Pupil Database | less complex | 116,963 | 116,963 | | 41.0% | | PAP WEB | |
| 1970 British Cohort Study: Forty-Two-Year Follow-Up | 2012 | GB | 42 | all children born in 1 week | none | not applicable | 9,764 | 8,437 | | 74.6% | | CASI | |
| EU Agency for Fundamental Rights: Violence Against Women Survey | 2012 | UK, EU | 18-74 | women | small user PAF | complex | 1,510 | - | | 36.9% | | SHOW | |

^a^ UK: United Kingdom, GB: Great Britain, ENG: England, ENG & WAL: England and Wales

^b^ PAF: postcode address file, HSCIC: Health and Social Care Information Centre

^c^ SHOW: face-to-face interview using show cards, TEL: telephone interview, PAP: paper-based self-completion questionnaire, CASI: face-to-face interview using computer-assisted self-completion module, WEB: online self-completion questionnaire.
